# Perinatal Birth Asphyxia Among Newborns at Jiblah Public Health Hospital in Ibb City, Yemen, During Six Years of Conflict and Its Predictive Factors: A Retrospective Cross-Sectional Study

**DOI:** 10.7759/cureus.54100

**Published:** 2024-02-12

**Authors:** Afaf Alsharif, Abdullah M Almatary, Faisal Ahmed, Mohamed Badheeb

**Affiliations:** 1 Gynecology, Jibla University for Medical and Health Sciences, Ibb, YEM; 2 General Surgery, Jibla University for Medical and Health Sciences, Ibb, YEM; 3 Urology, Ibb University, Ibb, YEM; 4 Internal Medicine, Yale New Haven Health, Bridgeport Hospital, Bridgeport, USA

**Keywords:** infant, hospital, yemen, ibb, predictors, developing country, birth asphyxia

## Abstract

Background: Birth asphyxia is a major cause of infant death across the world, especially in developing countries, where the issue is significantly underreported and underestimated, particularly in fragile and conflict-affected states.

Objective: The purpose of this study was to determine the prevalence and risk factors for birth asphyxia in women at Jiblah University Hospital in Ibb, Yemen, and its predictive factors throughout six years of conflict.

Method: We conducted a retrospective cross-sectional chart review and analysis of the birth database spanning from June 2013 to September 2019 at Jiblah University Hospital in Ibb, Yemen. We used APGAR (appearance, pulse, grimace, activity, and respiration) scores <7 at both the first- and fifth-minute post-delivery with umbilical cord arterial blood pH <7 (metabolic acidosis) and/or neurologic manifestations (seizures or an altered tone) within the first 24 hours of life to define birth asphyxia cases. Factors associated with birth asphyxia were analyzed using univariate and multivariate regression analysis with an odds ratio (OR) and 95% confidence interval (CI).

Results: A total of 5,193 neonates were delivered during the study period. The prevalence of birth asphyxia in 309 (6%) neonates. In a multivariate analysis, illiteracy (OR: 2.90; 95% CI: 0.98-8.41), referred mothers (OR: 3.04; 95% CI: 1.42-6.40), advanced maternal age (OR: 1.05; 95% CI: 1.02-1.07), home delivery (OR: 6.50; 95% CI: 3.09-12.57), prematurity (OR: 1.43; 95% CI: 1.05-1.93), and low birth weight (OR: 3.09; 95% CI: 1.93-4.93) were predictors for birth asphyxia and were statistically significant (p<0.05).

Conclusion: In this study, the prevalence of birth asphyxia was equivalent to that of other underdeveloped nations. However, continual attention and treatments are required to lower the risk of birth asphyxia. Illiteracy, referred mothers, advanced maternal age, home delivery, prematurity, and low birth weight were all predictors of birth asphyxia in this research. Most birth asphyxia factors mentioned in this study can be managed through effective prenatal, intrapartum, and postpartum care, as well as a strict following of national obstetrics and neonatal guidelines.

## Introduction

Perinatal mortality, despite witnessing a global decline, remains a significant health challenge [1]. While developed nations have reported decreased rates, conflict-afflicted regions exhibit mortality rates that are approximately 1.8-5 times higher [2,3]. This disparity is further highlighted by reports from the World Health Organization that indicate elevated perinatal mortality rates in Asia and Africa [4]. Notably, there is a prevailing underrecognition and underestimation of the profound implications that such mortality rates can have on maternal and fetal health [5].

Several factors have been identified as contributors to perinatal mortality. These include prematurity, multiple gestations, and maternal health-related factors, such as hypertension and diabetes. Additionally, fetal health concerns, ranging from anatomical abnormalities and infections to syndromic manifestations and birth asphyxia, play a major role in perinatal mortality incidence [6]. Among these, birth asphyxia stands out as a particularly salient risk factor that poses a threat to even-term infants, potentially leading to acute neurological impairment [7]. Generally, birth asphyxia indicates an impairment in either imitating or maintaining spontaneous breathing that subsequently leads to severe hypoxemia, with carbon dioxide retention leading to severe respiratory and metabolic acidosis with organ dysfunction [8].

The substantial impact of birth asphyxia is a heavy burden for families and healthcare systems alike. In developed countries, the occurrence of perinatal asphyxia is 2 per 1,000 births, while in developing countries with limited access to maternal and neonatal care, the rate is up to ten times higher. This is reported to result in perinatal death in 15% to 20% of cases and permanent neurological sequelae in up to 25% of survivors [9]. However, these figures might not capture the true magnitude in resource-deprived settings, such as Yemen. A recent report highlighted a staggering mortality rate of 89.3 per 1,000 in Yemen [10]. Conversely, a mortality rate of 5.4 per 1,000 live births was reported in the United States [11].

The war’s implications for Yemen's healthcare system appear to have a remarkable impact on perinatal mortality. Notably, poor access to healthcare facilities, along with inadequate perinatal care, might have worsened the perinatal mortality rates [5,12]. These findings shed light on a significant healthcare issue with detrimental consequences. The understanding of birth asphyxia determinants and predictors might help lower mortality rates. Thus, the present study endeavors to comprehensively review cases of birth asphyxia in Yemen, specifically focusing on the associated factors during its six-year military and geopolitical conflict.

## Materials and methods

Study setting

Jeblah Hospital is the main referral governmental hospital located in Jeblah, Ibb region, Yemen. It was built in 1965 and serves an Ibb region with a population of 772,000, according to the Central Statistics Agency [13]. Recently, this hospital has become an academic hospital for Jiblah University for Medical Sciences. The maternity ward had two beds in the delivery room and was staffed by a midwife or a nurse who attended deliveries and provided newborn resuscitation as needed. Additionally, a specific staff assigned to newborn care was also available. The pediatric unit was also located next to the maternity ward and received newborns who needed critical care.

Data sources and study design

Within a six-year period, we conducted a retrospective cross-sectional review and analysis of the birth database spanning from June 2013 to September 2019 at Jiblah University Hospital in Ibb, Yemen. All patients consented to and signed a consent form for the gathering of data from their medical records, as well as the publishing of their medical information, at the time of registration. The data, including demographic variables, socioeconomic indicators, and perinatal and intrapartum care, were methodically extracted from the hospital’s registry system. The study was conducted in accordance with the ethical principles outlined in the Declaration of Helsinki and received approval from the Jiblah University Ethics Research Committee (approval number: 167). The APGAR (appearance, pulse, grimace, activity, and respiration) score was utilized and for each component, a score of 0, 1, or 2 was given, with a score of <7 at both the first- and fifth-minute post-delivery with umbilical cord arterial blood pH <7 (metabolic acidosis) and/or neurologic involvement (seizures or an altered tone) within the first 24 hours of life, serving as the threshold for defining birth asphyxia based on the committee opinion of the American College of Obstetrics and Gynecology and the World Health Organization (WHO) (for cases: mothers with their newborns who were diagnosed with birth asphyxia, for controls: mothers with their newborns who were diagnosed without birth asphyxia) [14]. As for labor duration, any latent phase of the first stage of labor lasting beyond eight or 12 hours for multipara and primigravida, respectively, was classified as prolonged labor [15]. Additional definitions can be found in Table 1. Newborns born early preterm (under 28 weeks), stillbirths, or newborns with incomplete medical data were excluded from the study.

**Table 1:** Variable definitions APGAR: appearance, pulse, grimace, activity, and respiration

Condition	Definition/description
Birth asphyxia	Newborn with an APGAR score of <7 at the first and fifth minutes with umbilical cord arterial blood pH <7 (metabolic acidosis) and/or neurologic involvement (seizures or an altered tone) within the first 24 hours of life
Prolonged labor	A latent phase of the first stage exceeds 12 hours in primigravida or eight hours for multipara mothers
Premature membrane rupture	Rupture of the membrane of the amniotic sac and chorion occurs one hour before the onset of labor
Anemia during pregnancy	Maternal hemoglobin level <11 mg/dl
Gestational hypertension	Hypertension occurs after 22 weeks of gestation
Gestational age at birth	Estimated duration (in weeks) of pregnancy determined by health card report, last menstrual period, or the new Ballard assessment score on admission. Exclusion if none of these are obtainable
Low birth weight	Birth weight is less than 2,500 g (up to and including 2,499 g) [16]
Very low birth weight	Birth weight less than 1,500 g (up to and including 1,499 g)
Extremely low birth weight	Birth weight less than 1,000 g (up to and including 999 g)
Inborn neonates	Newborns born in Jiblah Hospital
Outborn neonates	Newborns born outside Jiblah Hospital (home delivery)

Data collection and outcome

Collected data included maternal age, socioeconomic status (low and moderate/high), place of residence (urban or rural), an education level (completed elementary school, completed high school, or illiterate), Khat-chewing status, history of smoking, history of diabetes mellitus, history of bleeding in pregnancy, history of premature rupture of membranes, history of iron-deficiency anemia, pre-eclampsia/eclampsia, place of delivery (at home or hospital), prolonged labor, antenatal care visit (no visits, one to three visits, and more than four visits), gestational age (more than 37 weeks and less than 37 weeks), parity (primipara and multipara), delivery mode (spontaneous vaginal delivery and instrumental, e.g., vacuum extraction and forceps; cesarean section), and booked status (referred and not referred mothers) were considered as maternal characteristics, while sex, birth weight, resuscitation, prematurity, congenital malformation, and prematurity were considered as neonatal characteristics. These collected factors were adapted from previous studies and contextualized based on their objectives [15,17]. The outcome is to determine the prevalence of birth asphyxia and its predictive factors.

Statistical analysis

Quantitative data was depicted as mean and standard variations, while categorical data was expressed in terms of counts and proportions. The normal distribution of the data was ascertained through the Smirnov-Kolmogorov test. Depending on the nature of the quantitative data, either the independent sample t-test or the Mann-Whitney U test was employed. For categorical data analysis, either the chi-square test or Fisher’s exact method was invoked. We utilized both univariate and multivariate logistic regression techniques to discern factors related to birth asphyxia. Any factor achieving a p-value of 0.2 or below in the univariate regression was subsequently examined in the multivariate regression. An odds ratio (OR) accompanied by a 95% confidence interval (CI) was determined. Factors that reached a p-value below 0.05 in a multivariate logistic analysis were deemed to have notable significance. All computations and analyses were executed using SPSS Statistics version 22.0 (IBM Corp. Released 2013. IBM SPSS Statistics for Windows, Version 22.0. Armonk, NY: IBM Corp.).

## Results

Sociodemographic and behavioral characteristics

The mean mother’s age was 24.5 ± 4.9 years, with a median of 24 years (interquartile range (IQR): 20.0, 28.0). The majority of mothers (75.8%) had antenatal care visits. Most of the mothers received some form of education (83.9%), with the majority having completed primary elementary education (73.4%). Most of them were from rural areas (58.5%) and had a low socioeconomic status (76.6%). Khat-chewing habits and smoking were presented in 25% and 10.1% of mothers, respectively (Table 2).

**Table 2:** Sociodemographic, behavioral, maternal health-related, and antepartum- and intrapartum-related variables of pregnant mothers

Variables	Subgroups	N (%)
Mother’s age (years)	Mean ± SD	24.5 ± 4.9 (range 18.0-37.0)
Socioeconomic status	Low	3,976 (76.6%)
Place of residency	Urban	2,155 (41.5%)
	Rural	3,038 (58.5%)
Educational level	Primary elementary school	3,814 (73.4%)
	High elementary school	542 (10.4%)
	Illiterate	837 (16.1%)
Habits	Khat chewing	1,298 (25.0%)
	Smoking history	525 (10.1%)
Illness during pregnancy	Diabetes	59 (1.1%)
	Bleeding during pregnancy	15 (0.3%)
	Iron-deficiency anemia	601 (11.6%)
	Pre-eclampsia	12 (0.2%)
Antenatal care visits	No visits	1,258 (24.2%)
	1-3 visits	2,006 (38.6%)
	≥4 visits	1,929 (37.1%)
Gestational age	≥37 weeks	1,053 (20.3%)
	<37 weeks	4,140 (79.7%)
Parity	Primipara	3,192 (61.5%)
	Multipara	2,001 (38.5%)
Place of delivery	Home	3,814 (73.4%)
	Hospital	1,379 (26.6%)
Delivery mode	Spontaneous vaginal delivery	3,986 (76.8%)
	Assisted vaginal delivery	439 (8.5%)
	Cesarean section	768 (14.8%)
Booked status	Not referred	5,069 (97.6%)
	Referred	124 (2.4%)

Maternal health-related variables

History of diabetes, bleeding during pregnancy, and iron-deficiency anemia were presented in 59 (1.1%), 15 (0.3%), and 601 (11.6%) cases, respectively. Referred mothers, premature rupture of membranes, and pre-eclampsia/eclampsia were presented in 124 (2.4%), 615 (11.8%), and 12 (0.2%) cases, respectively.

Antepartum- and intrapartum-related variables

The gestational age was <37 weeks in 4,140 (79.7%) cases, while the gestational age was ≥37 weeks in 1,053 (20.3%) cases, and most of the mothers (61.5%) were primiparous. Most mothers (73.4%) were delivered at home, and prolonged labor occurred in 889 (17.1%) cases. Spontaneous vaginal delivery was the most common method: 3,986 (76.8%), 439 (8.5%) required assisted vaginal delivery, and 768 (14.8%) were delivered via cesarean section.

Newborn characteristics

A total of 5,193 neonates were delivered, and 3,979 (76.6%) were male. Prematurity, congenital malformations (diagnosed prenatally), and birth trauma were presented in 173 (3.3%), 84 (1.6%), and 59 (1.1%) cases, respectively. Resuscitation was needed in 889 (17.1%) cases. Birth asphyxia occurred in 309 (6%) neonates (Table 3).

**Table 3:** Newborn characteristics

Variables	Subgroups	N (%)
Presentation of fetus	Cephalic	4,583 (88.3%)
	Breech or other presentation	610 (11.7%)
Fetal outcome	Twin	336 (6.5%)
	Single	4,857 (93.5%)
APGAR score fifth minute	Normal (between 7 and 10)	4,893 (94.2%)
	Abnormal (less than 7)	300 (5.8%)
Neonatal sex	Female	606 (11.7%)
	Male	4,587 (88.3%)
Resuscitation	-	889 (17.1%)
Birth trauma	-	59 (1.1%)
Malformation	-	84 (1.6%)
Low birth weight	-	173 (3.3%)

Birth asphyxia during the study period

The birth asphyxia was significantly reduced during the study period and was statistically significant (p=0.027) (Table 4).

**Table 4:** Neonatal characteristics in different years of study

Years	Total number	Alive	Dead	p-value
2013	1,261 (24.3%)	1,178 (24.1%)	83 (26.9%)	0.027
2014	891 (17.2%)	842 (17.2%)	49 (15.9%)	
2015	889 (17.1%)	839 (17.2%)	50 (16.2%)	
2016	734 (14.1%)	694 (14.2%)	40 (12.9%)	
2017	474 (9.1%)	431 (8.8%)	43 (13.9%)	
2018	485 (9.3%)	458 (9.4%)	27 (8.7%)	
2019	459 (8.8%)	442 (9.0%)	17 (5.5%)	

Factors associated with birth asphyxia

In univariate analysis, factors including antenatal care visits (OR: 1.49; 95% CI: 1.16-1.91, p=0.002), educational level (OR: 13.26; 95% CI: 10.35-17.09, p<0.001), referred mothers (OR: 6.59; 95% CI: 4.31-9.86, p=0.001), mother’s age (OR: 1.04; 95% CI: 1.02-1.07, p<0.001), current smoker (OR: 2.79; 95% CI: 2.09-3.69, p<0.001), premature rupture of membranes (OR: 7.57; 95% CI: 5.93-9.65, p<0.001), iron-deficiency anemia (OR: 2.60; 95% CI: 1.96-3.42, p<0.001), pre-eclampsia/eclampsia (OR: 7.99; 95% CI: 2.12-25.52, p=0.001), place of delivery (OR: 13.20; 95% CI: 9.99-17.72, p<0.001), mode of delivery (OR: 3.71; 95% CI: 2.39-5.63, p<0.001), prolonged labor (OR: 0.62; 95% CI: 0.43-0.88, p=0.009), gestational age (OR: 1.48; 95% CI: 1.14-1.92, p=0.003), fetal presentation (OR: 7.54; 95% CI: 5.91-9.62, p<0.001), number of fetus deliveries (OR: 0.62; 95% CI: 0.43-0.94, p=0.018), low birth weight (OR: 8.48; 95% CI: 5.97-11.92, p<0.001), and congenital malformation (OR: 5.21; 95% CI: 3.04-8.57, p<0.001) were associated with birth asphyxia and were statistically significant (Table 5).

**Table 5:** Univariate and multivariate analysis of factors associated with birth asphyxia OR: odds ratio, CI: confidence interval In bivariate (sample t-test or Mann–Whitney U test for quantitative data and chi-square test or Fisher’s exact test for categorical data) and multivariable analysis (backward logistic regression), * and ** denote statistically significant associations at p=0.05, respectively

Variables	Subgroup	No birth asphyxia (%)	Birth asphyxia N (%)	Univariate	p-value	Multivariate	p-value
				OR (95% CI)		OR (95% CI)	
Antenatal care visit	Yes	3,724 (94.6)	211 (5.4)	Reference group	0.002^*^	Reference group	0.248
	No	1,160 (92.2)	98 (7.8)	1.49 (1.16-1.91)		1.21 (0.87-1.68)	
Educational level	Educated	4,252 (97.6)	104 (2.4)	Reference group	<0.001^*^	Reference group	0.011^**^
	Illiterate	632 (75.5)	205 (24.5)	13.26 (10.35-17.09)		2.90 (0.98-8.41)	
Booked status	Not referred	4,794 (94.6)	275 (5.4)	Reference group	0.001^*^	Reference group	0.004^**^
	Referred	90 (72.6)	34 (27.4)	6.59 (4.31-9.86)		3.04 (1.42-6.40)	
Mother's age (year)	Mean (SD)	24.4 (4.8)	25.5 (5.9)	1.04 (1.02-1.07)	<0.001^*^	1.05 (1.02-1.07)	<0.001^**^
Current smoking history	No	4,428 (94.9)	240 (5.1)	Reference group	<0.001^*^	Reference group	0.548
	Yes	456 (86.9)	69 (13.1)	2.79 (2.09-3.69)		0.81 (0.40-1.63)	
Premature rupture of membranes	No	4,408 (96.3)	170 (3.7)	Reference group	<0.001^*^	Reference group	0.700
	Yes	476 (77.4)	139 (22.6)	7.57 (5.93-9.65)		0.64 (0.03-4.82)	
History of iron-deficiency anemia	No	4,357 (94.9)	235 (5.1)	Reference group	<0.001^*^	Reference group	0.235
	Yes	527 (87.7)	74 (12.3)	2.60 (1.96-3.42)		1.48 (0.76-2.80)	
Pre-eclampsia/eclampsia	No	4,876 (94.1)	305 (5.9)	Reference group	0.001^*^	Reference group	0.150
	Yes	8 (66.7)	4 (33.3)	7.99 (2.12-25.52)		3.22 (0.63-16.26)	
Place of delivery	Home	3,752 (98.4)	62 (1.6)	Reference group	<0.001^*^	Reference group	<0.001^**^
	Hospital	1,132 (82.1)	247 (17.9)	13.20 (9.99-17.72)		6.50 (3.09-12.57)	
Prolonged labor	No	4,031 (93.7)	273 (6.3)	Reference group	0.009^*^	Reference group	<0.001^**^
	Yes	853 (96.0)	36 (4.0)	0.62 (0.43-0.88)		0.18 (0.12-0.27)	
Gestational age	≥37 weeks	3,914 (94.5)	226 (5.5)	Reference group	0.003^*^	Reference group	0.021^**^
	<37 weeks	970 (92.1)	83 (7.9)	1.48 (1.14-1.92)		1.43 (1.05-1.93)	
Delivery mode	Vaginally	3,906 (98.0)	80 (2.0)	Reference group		Reference group	
	Vaginally with assist	408 (92.9)	31 (7.1)	3.71 (2.39-5.63)	<0.001^*^	0.66 (0.32-1.44)	0.267
	Cesarean section	570 (74.2)	198 (25.8)	16.96 (12.95-22.41)	<0.001^*^	1.38 (0.62-3.44)	0.454
Fetal presentation	Cephalic	4,412 (96.3)	171 (3.7)	Reference group	<0.001^*^	Reference group	0.807
	Breech	472 (77.4)	138 (22.6)	7.54 (5.91-9.62)		1.33 (0.17-27.20)	
Number of fetuses	Twin	306 (91.1)	30 (8.9)	Reference group	0.018^*^	Reference group	0.160
	Single	4,578 (94.3)	279 (5.7)	0.62 (0.43-0.94)		0.69 (0.41-1.17)	
Low birth weight	No	4,765 (94.9)	255 (5.1)	Reference group	<0.001^*^	Reference group	<0.001^**^
	Yes	119 (68.8)	54 (31.2)	8.48 (5.97-11.92)		3.09 (1.93-4.93)	
Malformation	No	4,820 (94.3)	289 (5.7)	Reference group	<0.001^*^	Reference group	0.153
	Yes	64 (76.2)	20 (23.8)	5.21 (3.04-8.57)		0.5403 (0.22-1.29)	

However, in a multivariate logistic regression analysis, illiteracy (OR: 2.90; 95% CI: 0.98-8.41), referred mothers (OR: 3.04; 95% CI: 1.42-6.40), advanced maternal age (OR: 1.05; 95% CI: 1.02-1.07), home delivery (OR: 6.50; 95% CI: 3.09-12.57), lower gestational age (OR: 1.43; 95% CI: 1.05-1.93), and prematurity (OR: 3.09; 95% CI: 1.93-4.93) were predictors for birth asphyxia and were statistically significant (p<0.05) (Table 5).

## Discussion

The incidence of birth asphyxia varies significantly among regions, with a disproportionate impact on low- and middle-income countries, wherein the ramifications of birth asphyxia are ubiquitously evident, irrespective of the birth geography [18]. Birth asphyxia rates in underdeveloped nations are exceptionally high, ranging from 4.6 per 1,000 to 26 per 1,000 births, with case fatality rates of more than 40% [19]. In our study, we observed a birth asphyxia rate of 6%. This trajectory, while demonstrating a decline over sequential years, manifests as a noticeable spike concurrent with national conflict. These findings are somewhat lower than a prior report that showed a rate of 15% [20]. Such disparities might be extrapolated from the variances in conflict intensity within the particular region. In comparison, the estimated birth asphyxia in southern Ethiopia was 15.1% [21]. However, these numbers should be interpreted cautiously, as variations in settings and populations within each country can lead to differing mortality rates, as shown in previous studies [19-21].

Several factors have been identified to be associated with birth asphyxia, including antenatal care and maternal education level [18]. While 83.9% of mothers in our study have received some form of formal education, a preponderant majority, quantified at 73.4%, plateaued at the elementary level. The impact of educational level manifested itself in our univariate and multivariate analyses concerning its association with birth asphyxia. Indeed, noticeable variations in such a variable were reported differently. For instance, reports from Pakistan [22], Nepal [23], and Cameroon [24] revealed a higher incidence of birth asphyxia among illiterate mothers; however, a report from Ethiopia [25] did not identify any significant association. Such disparities might be attributed to latent variables, such as rural habitation. Moreover, the demarcation between formal and informal education does not necessarily delineate literacy levels accurately.

We also identified home delivery as being associated with birth asphyxia. Such correlations, while pertinent to Yemen, are not geographically exclusive. A population-based cohort study in the US found that out-of-hospital deliveries were linked to higher perinatal mortality compared to in-hospital deliveries. However, this might be underestimated because many cases included planned deliveries, often with certified midwives present at the time of delivery [26]. These findings were not available in our hospital registry; however, we believe that in a country like Yemen, where home deliveries are common, the availability of birth centers or trained village workers may help in decreasing, even partially, the risk of complicated deliveries. This could also aid in ensuring an earlier hospital transition, where further medical interventions can be provided to mitigate the risk of birth asphyxia. Such an approach has been implemented in India and has been shown to be associated with reduced mortality [27]. Furthermore, our analysis showed that lower gestational age, prematurity, referred mothers, and advanced maternal age were associated with birth asphyxia, while few retrospective studies have shown variable results; the majority of pooling studies were consistent with our findings [18,28].

Counterintuitively, while our univariate analysis indicated prenatal care, antepartum hemorrhage, pre-eclampsia, eclampsia, prolonged rupture of membranes, and prolonged (obstructed) labor to be associated with birth asphyxia, the multivariate analysis precludes the statistical significance. Although such findings do not align with major studies that have shown a strong association between these factors and birth asphyxia [29], they have been reported, with no substantial association, in multiple singular retrospective studies [25,30,31]. This may be attributed to the variability of the studies’ settings and the heterogeneity of the patient population, which may render few unadjusted variables affecting the overall association. Furthermore, the smaller number of cases with these factors within each study may vary as well, limiting the power of the study. Lastly, the retrospective nature of these studies, including ours, increases the risk of recall and attrition bias. Indeed, we strongly believe that a more comprehensive population-based study should be conducted to identify each risk factor and indicate the possible relationship to birth asphyxia.

In this study, preterm newborns had a 1.43 times higher risk of asphyxia than mature babies. This finding is consistent with prior investigations, such as Tasew et al. in Ethiopia and Goldenberg et al. [30,31]. This might stem from the vulnerability of preterm newborns to ischemic brain insults due to insufficient blood-brain barrier development. In addition, the possible concomitant lung immaturity can lead to additional hypoxic injury to the brain. We also observed a threefold greater risk of birth asphyxia among newborns with low birth weight compared with those with normal birth weight. Nevertheless, our multivariate analysis did not substantiate these observations. These outcomes are similar to reports undertaken in central Ethiopia and Gondar [15,17]. Notably, higher rates of maternal comorbidities, including hypertension and diabetes mellitus, were reported among low-birth-weight newborns, which may independently increase the risk of birth asphyxia [15]. In a review by Lu et al. [32], prenatal care did not exhibit a significant role in preventing low-birth rats. Nevertheless, the quality of healthcare delivery might influence such findings. Redding et al. utilized a community model that targets mothers at risk and provides prenatal tracking and follow-up, which was associated with a lower risk of low birth weight [33]. Furthermore, the optimization of medical conditions during pregnancy might be influential in the prevention of low birth weight. This might be presumed from prior reviews that showed that pregnancies complicated by hypertension were associated with higher rates of low birth weight [34].

Birth asphyxia was significantly reduced during the study period and was statistically significant. This may be due to the financial support provided by several organizations in facilitating and funding our hospital in recent years [35]. In fact, with the support of WHO and other organizations in facilitating and funding our hospital, the rate of admission and offering several maternal services played a role in improving our hospital, which will be investigated in the future.

Study limitations

This study is limited by its retrospective monocentric design, which makes the collected data susceptible to misclassification and recall bias. Additionally, relying on the hospital registry could lead to documentation issues, including incomplete data and lost files. The selection of the study retrospective is also potentially biased, as it may not be representative of the general population. Our result needs to be validated in a large prospective multicenter study with strict criteria for birth asphyxia definition and detailed recording. Although the study encompassed patients from both rural and urban backgrounds, there is a concern that urban patients who presented to the hospital had distinct backgrounds (e.g., higher education). This might have resulted in certain cases going unrecognized.

## Conclusions

In this study, the prevalence of birth asphyxia was equivalent to that of other underdeveloped nations. However, continual attention and treatments are required to lower the risk of birth asphyxia. Illiteracy, referred mothers, advanced maternal age, home delivery, prematurity, and low birth weight were all predictors of birth asphyxia in this research. Most birth asphyxia factors mentioned in this study can be managed through effective prenatal, intrapartum, and postpartum care, as well as a strict following of national obstetrics and neonatal guidelines.
